# Turning the spotlight on bone marrow adipocytes in haematological malignancy and non‐malignant conditions

**DOI:** 10.1111/bjh.18748

**Published:** 2023-04-17

**Authors:** Michael J. Austin, Foteini Kalampalika, William P. Cawthorn, Bela Patel

**Affiliations:** ^1^ Barts Cancer Institute, Centre for Haemato‐Oncology Queen Mary University of London London UK; ^2^ BHF/University Centre for Cardiovascular Science, Edinburgh Bioquarter University of Edinburgh Edinburgh UK

**Keywords:** adipocytes, haematological malignancies, leukaemia, minimal residual disease, multiple myeloma

## Abstract

Whilst bone marrow adipocytes (BMAd) have long been appreciated by clinical haemato‐pathologists, it is only relatively recently, in the face of emerging data, that the adipocytic niche has come under the watchful eye of biologists. There is now mounting evidence to suggest that BMAds are not just a simple structural entity of bone marrow microenvironments but a bona fide driver of physio‐ and pathophysiological processes relevant to multiple aspects of health and disease. Whilst the truly multifaceted nature of BMAds has only just begun to emerge, paradigms have shifted already for normal, malignant and non‐malignant haemopoiesis incorporating a view of adipocyte regulation. Major efforts are ongoing, to delineate the routes by which BMAds participate in health and disease with a final aim of achieving clinical tractability. This review summarises the emerging role of BMAds across the spectrum of normal and pathological haematological conditions with a particular focus on its impact on cancer therapy.

**Abbreviations**
AA: aplastic anaemia
ALL: acute lymphoblastic leukaemia
AML: acute myeloid leukaemia
BAT: brown adipose tissue
BM: bone marrow
BMAd: bone marrow adipocyte
BMAT: bone marrow adipose tissue
BMME: Bone Marrow Microenvironment
cBMAd: constitutive bone marrow adipocyte
FA: fatty acid
GAT: gonadal adipose tissue
GDF‐15: growth differentiation factor 15
HSC: Haematopoietic stem cell
LEPR: leptin receptor
LSC: leukaemia stem cell
MM: Multiple Myeloma
MSC: Mesenchymal Stromal Cells
rBMAd: regulated bone marrow adipocyte
Sc‐RNAseq: single cell RNA sequencing sc‐RNAseq
SCF: stem cell factor
SMM: smouldering myeloma
SSC: skeletal stem cells
TME: tumour microenvironment
WAT: white adipose tissue

## INTRODUCTION

**FIGURE 1:**
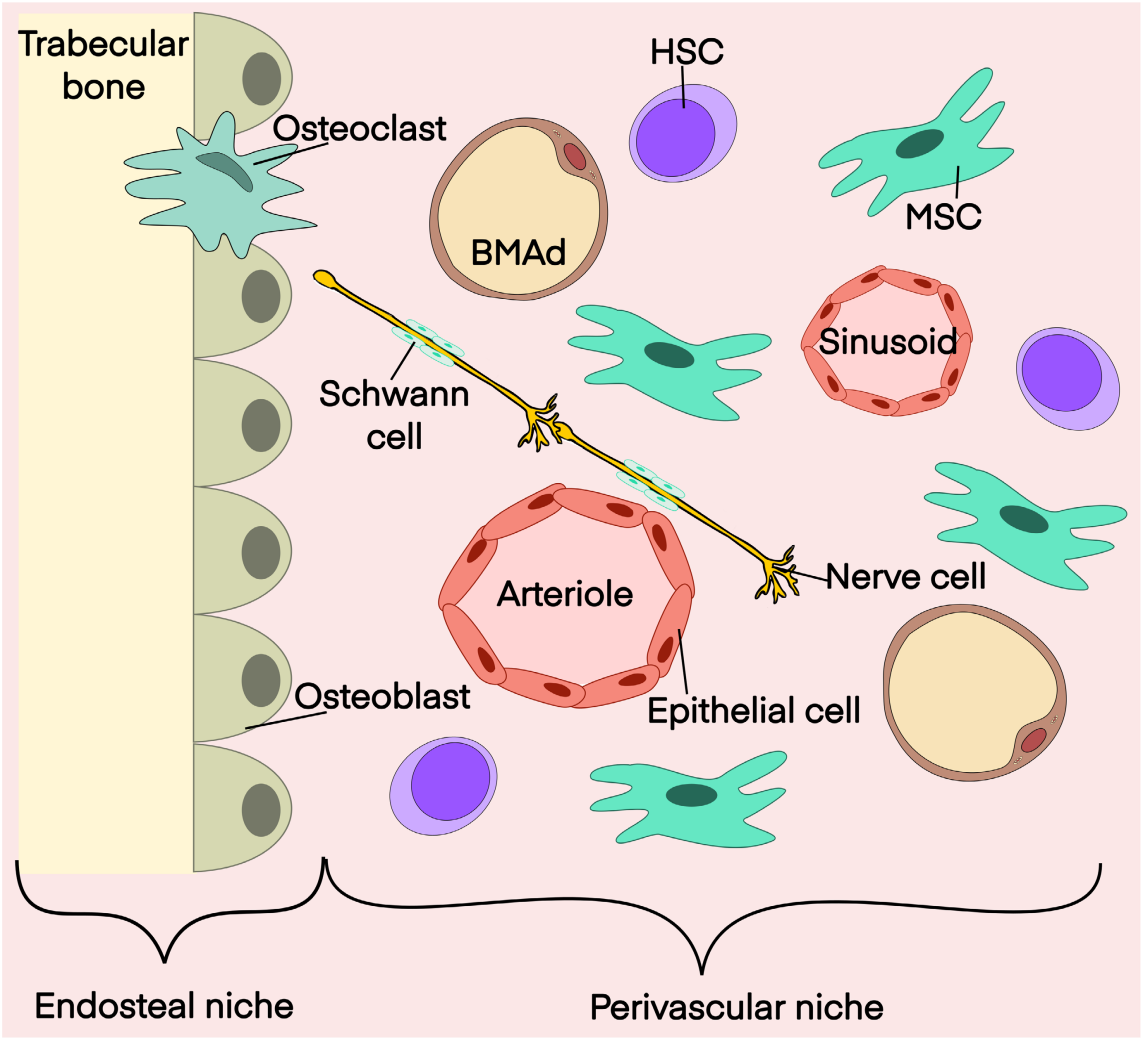
Schematic of the healthy BMME. Major tissue participants of BMME involved in blood cell production and HSC homeostasis. BMME, Bone marrow microenvironment; MSC, Mesenchymal stromal cell; HSC, Haematopoietic stem cell; BMAd, Bone Marrow adipocyte.

The bone marrow (BM) niche is composed of a heterogeneous mixture of haematopoietic stem cells (HSCs) and their progeny as well as a multitude of non‐haematopoietic cells, collectively referred to as a BM microenvironment (BMME). The major components of BME are mesenchymal stromal cells (MSC), osteoblasts, endothelial cells, smooth muscle cells, non‐myelinating Schwann cells, nerve cells as well as BM adipocytes (BMAd) (Figure 1). Together they constitute a highly elaborate network that contributes in a precise and rapid manner to HSC function and homeostasis under changing physiological contexts.^1^, ^2^, ^3^ In malignant syndromes, both those of haematological origin^4^ and secondary metastatic,^5^, ^6^ the BM microenvironemnt (BMME) is co‐opted to facilitate tumour‐cell proliferation and survival at the expense of its primary HSC‐supportive function. Consequently, intense efforts are underway to identify which participants and mechanisms of BMME mediate critical tumour‐enabling effects with the prospect that such insights will yield new therapeutic avenues. An emerging entity here is BM adipose tissue (BMAT), once regarded as a simple lipid storage structure, paradigms for these components are shifting to a more complex definition as a key governing principle in haematopoietic health and disease that warrants further exploration.

One of the first insights into this niche came as early as the late 19th century when in 1898, the presence and potential significance of BMAds were first described whilst studying the effect of arsenic in the BM and blood of rabbits.^7^ They noted that the ‘fat cells’ present in the BM were significantly reduced in numbers and appeared atrophied after treatment with arsenic when compared to controls, highlighting the principle that BMAd tissues are not biologically inert. More than 120 years since this initial observation, and only as recently as the last decade, BMAds have come under the sharp focus of basic and translational research as a major biological concept. This review will discuss the results and highlight the latest developments in our understanding in the BMAd field across both normal and pathological haematological contexts particularly focusing on the contribution of these tissues to altering the response to cancer treatment.

## PHYSIOLOGY OF BMAT

BMAT represents over 10% of total fat mass in lean adult humans, with a typical anatomical distribution that arises over a course of well‐characterised postnatal development.^8^ In mammals, BMAds are mostly absent at birth and begin to form perinatally in distal portions of the long bones. During puberty, there is a rapid expansion of BMAT, particularly in the appendicular skeleton, whilst red haematopoietic marrow continues to predominate in the axial bones. Notably, this BMAT expansion coincides with an increased bone acquisition, demonstrating that increased BM adiposity is not always associated with bone loss. Thus, in healthy 25‐year‐olds BMAT is a major anatomical component that accounts for approximately 70% of total BM volume.^9^

**FIGURE 2:**
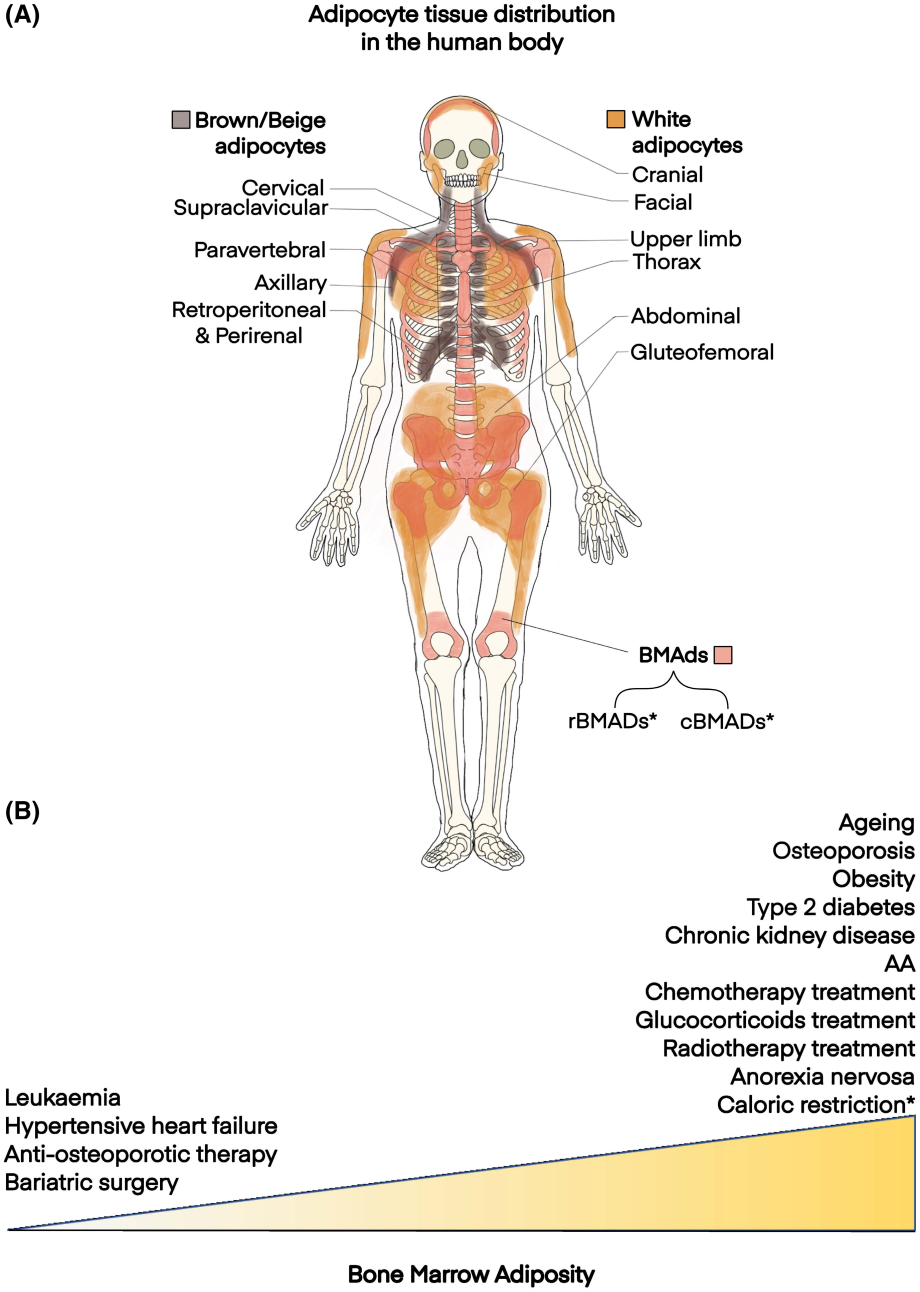
Human and murine adipocytic tissues. (A) Adipocyte tissue distribution in the human body. (B) Dynamics of Bone Marrow Adiposity under during different physiological and pathophysiological contexts. *primarily defined in murine models. BMAd, Bone marrow adipocyte; cBMAd, constitutive bone marrow adipocyte; rBMAd, regulated bone marrow adipocyte; AA, Aplastic anaemia.

BMAT further accumulates with ageing and in diverse clinical conditions, including osteoporosis; obesity and type 2 diabetes; chronic kidney disease; aplastic anaemia (AA); and in response to glucocorticoids, radiotherapy or chemotherapy.^10^, ^11^ Conversely, BM adiposity declines in various leukaemias, hypertensive heart failure and in response to anti‐osteoporotic therapies or bariatric surgery (reviewed in Reference [8]). Unlike the two most‐recognised adipose subtypes, white and brown adipose tissue (WAT and BAT), BMAT also increases during anorexia nervosa and in animal models of caloric restriction. This suggests that BMAT is developmentally and functionally distinct to WAT and BAT. Consistent with this, research in animal models confirms that BMAd progenitor cells differ from those for white or brown adipocytes,^1^, ^12^, ^13^ whilst other studies, including in humans, have shown that BMAT is molecularly and metabolically distinct to WAT and BAT.^14^, ^15^, ^16^, ^17^ Together, these observations identify BMAT as a third major, distinct subtype of adipose tissue that is altered in diverse pathophysiological contexts (Figure 2A,B).

**FIGURE 3:**
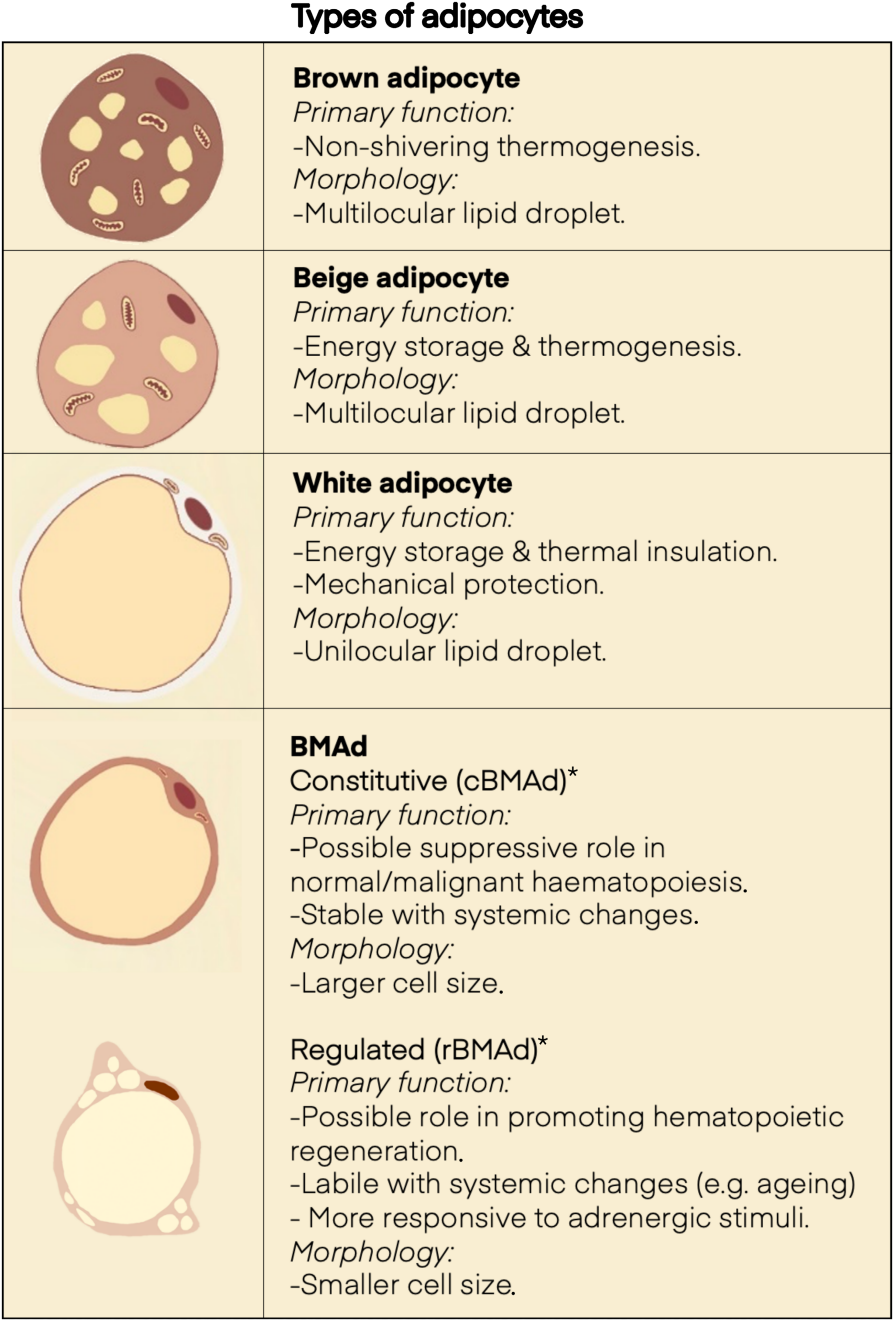
Types of adipocytes. Summary of the major morphological and functional differences between distinct adipocyte tissue subtypes. *primarily defined in murine models.

One important nuance is that not all BMAds are the same: they are proposed to exist in two broad subtypes, ‘constitutive’ and ‘regulated’ (cBMAds and rBMAds), which differ in anatomical distribution and in their developmental, morphological, molecular and functional characteristics (Figure 3). The cBMAds predominate in the distal appendicular skeleton, form early in development, are relatively resistant to expansion and breakdown in pathophysiological contexts and exist as contiguous groups of adipocytes, similar to those within WAT. In contrast, rBMAds accumulate in the axial and proximal appendicular skeleton during ageing and other clinical contexts (e.g. ageing anorexia, irradiation and hypoglycaemic thiazolidinediones) existing as discrete cells or as small clusters of adipocytes interspersed amongst haematopoietic marrow.^14^, ^18^, ^19^, ^20^ Thus, rBMAds are highly sensitive to a range of systemic challenges. Whilst it is important to note such subtypes of BMAds are primarily characterised in mice and not humans, the principle that within BMATs there is heterogeneity will likely have implications for defining the precise role contribution of these tissues in health and in disease linked states.

What causes BMAd accumulation or depletion in the above pathophysiological conditions, and do BMAds directly influence human health and disease? These are two key questions that are beginning to be resolved. BM adiposity typically shows an inverse association with bone mass and haematopoietic output, and therefore, the roles of BMAds in ageing‐associated bone loss and haematological disorders have received particular attention. One concept is that BMAds secrete endocrine and paracrine factors to directly suppress bone formation, promote bone loss and inhibit haematopoiesis.^8^, ^21^ For example, BMAds may suppress osteoblasts via release of cytokines, lipids and adipogenic RNAs that disrupt osteoblast function.^21^, ^22^, ^23^, ^24^ Conversely, BMAds or their progenitor cells can secrete RANKL, CXCL1 and CXCL2 to stimulate osteoclast activity and thereby promote bone loss.^25^, ^26^, ^27^, ^28^, ^29^ BMAds’ latter effect may also facilitate the spread of skeletal tumours, including metastases from the breast or prostate.^26^, ^30^ Finally, BMAd production of DPP‐4 has been implicated with impaired fracture healing, at least in mice.^31^ Thus, BMAds secrete a myriad of factors that may modulate bone remodelling and skeletal diseases.

## BMAd AND HAEMATOPOIESIS

Whilst observations such as age‐dependant decline of haematopoietic function and BMAd accumulation in AA conditions support the concept that BMAds suppress haematopoiesis; functional studies addressing this possibility have yielded inconsistent results. In mice, adipocyte‐rich BM niches in tail vertebrae (vs. adipocyte‐poor thoracic vertebrae) correlate negatively with HSC numbers and haematopoietic output^32^ suggesting a negative role of BMAds in haematopoietic regulation. Substantiating this notion, fatless A‐ZIP/F1 mice, which are genetically devoid of adipocytes, demonstrate rescued haematopoietic output in tail‐BM both in steady‐state and after radiation injury.^32^ Similarly, pharmacological approaches that prevent new BMAd formation after irradiation or chemotherapy promote haematopoietic recovery.^32^, ^33^ Together with reports that co‐transplantation of murine adipocytic lineage‐committed cells impairs the HSC haematopoietic reconstitution potential in vivo^31^ and repressive outcomes on granulopoiesis,^34^ this has been interpreted to reflect a negative effect of BMAd on haematopoiesis. In contrast, other research shows that cultured human BMAds can support HSC maintenance, possibly through secretion of pro‐haematopoietic factors.^35^ Indeed, Zhou et al. found, genetic deletion of BMAds in mice suppresses haematopoietic regeneration in the long bones after irradiation or 5‐fluorouracil treatment but enhances regeneration within BMAd‐rich tail vertebrae.^36^ These discrepancies may reflect differences between BMAd subtypes; namely, that cBMAds (tail‐BM) suppress regeneration whereas rBMAds exert pro‐regenerative effects. Importantly, the implication here is that BM adipogenesis may aid stress haemopoiesis,^36^ which is consistent with clinical scenarios in HSC transplantation where irradiation is expected to increase BMAd number^11^ concomitant with HSC proliferation.

Mechanistically, BMAds may exert effects on haematopoietic function by secreting paracrine factors and by acting as a local energy source. A notable example of the former is stem cell factor (SCF), a major HSC‐linked niche factor. Genetic deletion of SCF in BMAds or BMAd precursors or depletion of its main cellular source; the Leptin receptor (LEPR) expressing mesenchymal stroma (but not osteoblasts, endothelial cells or haematopoietic cells), suppresses haematopoietic regeneration in mice following irradiation or chemotherapy^36^ and was shown to decrease HSC and myeloid precursor populations under physiological conditions.^37^ This suggests that BMAds can support haematopoiesis by secreting SCF.

Additional mechanisms of BMAd involvement in haematopoiesis include secretion of adiponectin^38^ and leptin. LEPR is expressed in normal CD34+ expressing progenitor cells and HSC populations^39^ and human BMAds constitute an important source of Leptin.^40^ Functional effects of Leptin on haematopoiesis include; increased proliferation of human HSCs^39^ and murine myelocytic progenitors in addition to a synergistic effect with SCF to enhance the proliferation of primitive haematopoietic progenitors.^41^ In murine models, adiponectin is expressed abundantly in whole BM stromal cell populations and has been shown to exert pro‐proliferative effects on HSCs in vitro, with accompanying enhancement of haematopoietic reconstitution capacity upon transplantation into lethally irradiated mice.^42^

Ageing in humans is associated with increased BM fat expansion, prompting exploration for a functional connection with age‐related haematological decline.^43^ Supporting such a link is the observation that BMAds negatively regulate B lymphopoiesis and skew towards myelopoiesis,^44^, ^45^, ^46^ which may account for the phenomenon of age‐related decline in B‐lymphocyte generation. This described limitation in B‐lymphocyte generation may occur via a soluble adipocyte‐derived factor, which acts to arrest Common Lymphoid Progenitor to pre/pro‐B transitions in human HSC in vitro.^47^ A further indirect mechanism is through the inflammatory microenvironment induced by BMAT, and Il‐1β production by inflammatory myeloid cell constituents.^48^

On a metabolic level, BMAds may further influence haematopoiesis as a source of fatty acids (FA), which adipocytes release following lipolysis of their internal triglyceride stores. Although BMAds resist lipolysis in response to typical systemic cues, such as fasting or sympathetic activation,^15^, ^20^ they can respond to local lipolytic signals within the BM niche. The haematological role of BMAd lipolysis in other contexts has remained unresolved, but a recent study by Li et al. provides some answers; they prevented BMAd lipolysis using a novel mouse model allowing specific transgenic targeting of BMAds^49^ and revealed that BMAd lipolysis is required to support myelopoiesis during caloric restriction, suggesting a key role for BMAd lipolysis in haematopoietic regulation during altered metabolic states. One limitation is that these observations are largely based on mouse models; thus, whether BMAds directly modulate haematopoiesis in humans, both in physiological and clinical contexts, remains to be firmly established.

The above observations highlight the impact of BMAd‐secreted factors, including energy substrates, in the relationship between BMAds, haematopoietic regulation and bone health. However, BMAd progenitors likely also play an important role. Several recent studies show that ageing alters the composition and function of skeletal stem cells (SSCs), the BM‐resident progenitors that give rise to BMAds, osteoblasts, chondrocytes and the stromal cells that support haematopoiesis.^50^ For example, in both mice and humans ageing is associated with decreased osteogenic but increased adipogenic capacity of SSCs,^31^, ^51^, ^52^, ^53^, ^54^ with some studies finding that ageing gives rise to a distinct population of adipogenic SSCs.^12^ In mice, the adipogenic SSCs directly impair fracture repair and haematopoietic reconstitution,^31^ suggesting that BMAd accumulation may directly contribute to age‐associated bone loss and haematological dysfunction. However, another recent study found that blocking BMAT formation in mice does not prevent bone loss with ageing, suggesting that BMAT expansion is not necessary for this effect.^55^ Thus, the contribution of BMAds to age‐associated bone loss remains to be established.

## SOURCE OF BMAd IN THE BM NICHE

BMAds arise through terminal differentiation of BM‐resident MSC (BM‐MSC) populations via coordinated programmes that may become perturbed in health and disease contexts.^56^, ^57^, ^58^

Human BM‐MSCs are functionally defined as plastic adherent populations that  express markers CD73, CD90 and CD105 and lack expression of haematopoietic cell and progeny markers such as CD45, CD34, CD14 or CD11b, CD79α or CD19 and HLA‐DR with a capacity for in vitro  multilineage differentiation (adipocytes, osteoblasts and chondrocytes).^56^ The advent of single‐cell RNA sequencing (sc‐RNAseq) studies, has enabled further dissection of these BM‐MSCs at least in mice. Such studies have helped to resolve the identity of a discrete and distinct adipocyte progenitor beyond previous concepts.^1^, ^13^, ^59^ In murine BM, LEPR expression denotes cells that are the main source of adipocyte and osteoblast differentiation but chondrocytic differentiation is more limited.^60^ Sc‐RNAseq defines that within this LEPR+ subset, *Lpl* ^high^ and *Mgp* ^high^   in addition to ESM1 staining^1^, ^13^, ^36^, ^60^ likely specify a restricted adipocyte progenitor population. Recent work by Zhong et al. identified a novel adipoprogenitor BM population in mouse models using labelled BM mesenchymal lineage cells,^61^ which they term adipocyte lineage precursors (MALPS).^62^ MALPS were identified computationally by sc‐RNAseq and validated functionally and were shown to express common adipocyte markers, but no lipid droplets.

**FIGURE 4:**
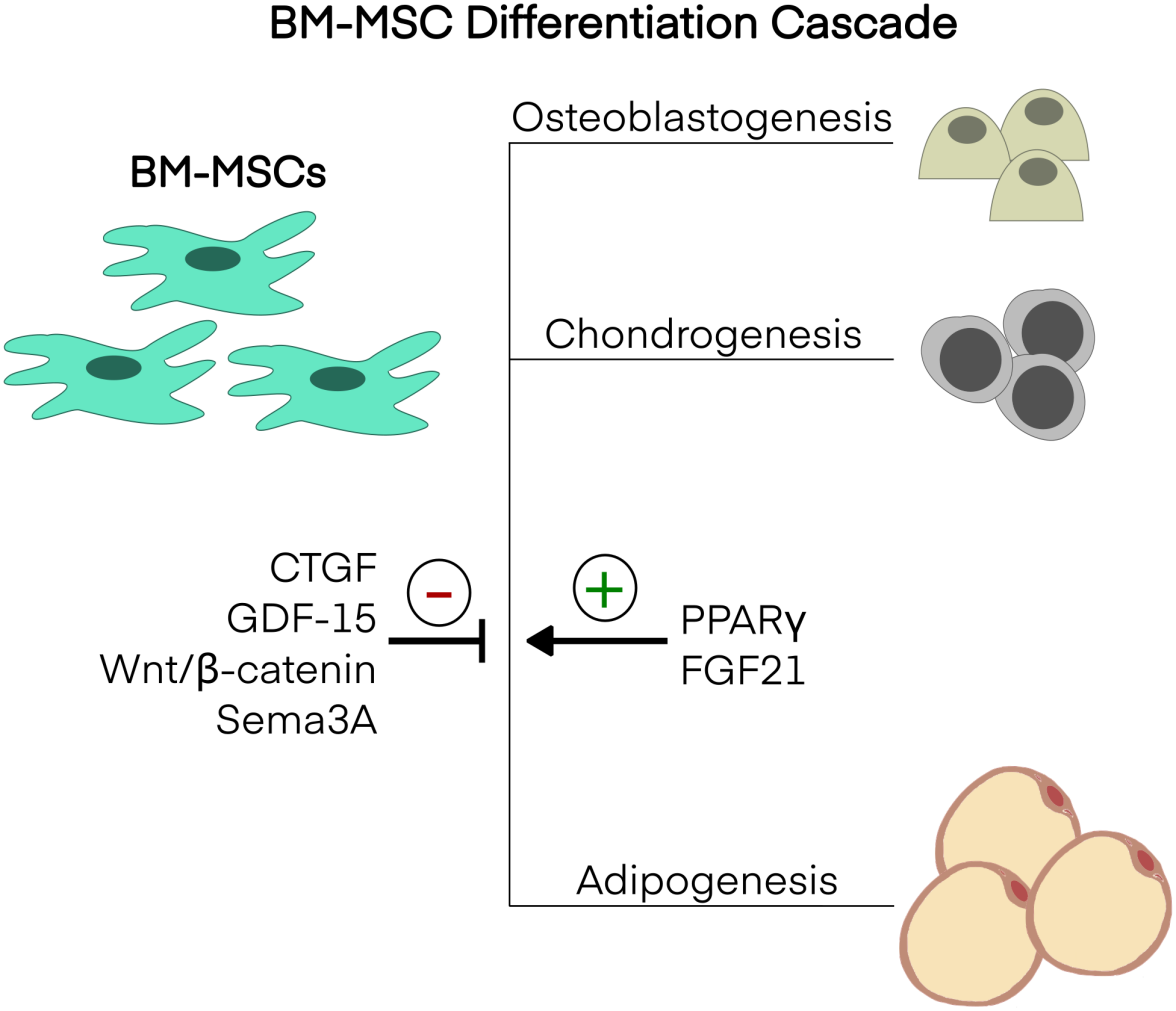
A Schematic of BM‐MSC differentiation outcomes showing several; positive and negative regulators of BM adipogenesis. BM, Bone marrow; MSC, Mesenchymal stem cell; CTGF, Connective tissue growth factor; GDF‐15, Growth/differentiation factor‐15; wnt, Wingless/Integrated; Sema3A, Semaphorin 3APPARγ, Peroxisome proliferator‐activated receptor gamma; FGF21, Fibroblast factor 21.

Along with efforts to resolve the identity of adipocytic precursor populations, pathways regulating the finely tuned balance between adipogenesis and osteogenesis are also important to resolving the potential molecular and cellular basis for altered adipocyte population dynamics associated with health, ageing and disease (Figures 2B and 4). Positive inducers of adipocytic differentiation in MSC include activation of the nuclear receptor PPARγ and fibroblast factor 21, which may also correspondingly suppress osteoblastic differentiation. Conversely, Wnt/β‐catenin and Semaphorin 3A signalling pathways have the opposite outcome. Connective tissue growth factor suppresses adipogenic outcomes/capabilities in MSC and similar outcomes are reported with cytokine growth differentiation factor 15 (GDF‐15).^63^, ^64^

## ROLE OF BMAd IN HAEMATOLOGICAL MALIGNANCY

**FIGURE 5:**
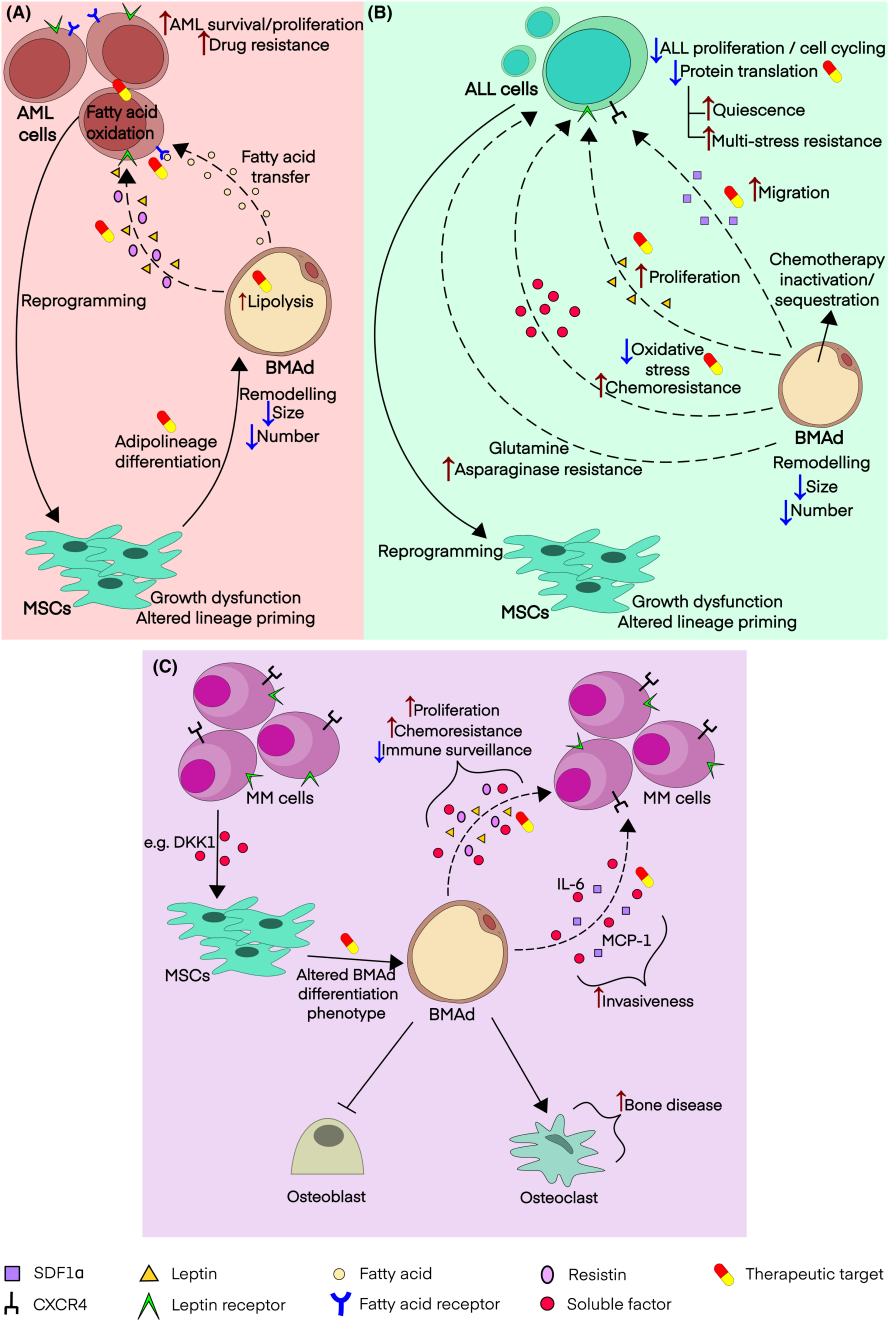
Summary of the main modes of cross‐talk between BMAd and haematological malignancies. (A) Acute myeloid leukaemia, (B) Acute lymphoblastic leukaemia. AML, Acute myeloid leukaemia; MSCs, Mesenchymal stem cells; BMAd, Bone marrow adipocyte; SDF1α, stromal cell‐derived factor 1 alpha; AML, Acute lymphoblastic leukaemia. (C) Summary of the main modes of cross‐talk between BMAd and Multiple Myeloma. MSCs, Mesenchymal stem cells; BMAd, Bone marrow adipocyte; MM, Multiple myeloma; DKK1, Dickkopf WNT signalling pathway Inhibitor 1; IL‐6, Interleukin 6; MCP‐1, Monocyte chemoattractant protein‐1.

Whilst adipocytes are well‐recognised in the tumour microenvironment (TME) of many solid cancers,^65^ a distinct role of BMAT has also begun to emerge for BM‐resident malignancies. Within existing descriptions, the role of BMAds in haematological malignancy is best delineated in acute myeloid leukaemia (AML), acute lymphoblastic leukaemia (ALL) (Figure 5A,B) and Multiple Myeloma (MM) (Figure 5C). In this section, what is known in these conditions will be reviewed as well as emerging data in other disease contexts. An important caveat is that biological models have not been restricted to the BMAd itself but may also include other adipocyte tissue contexts, including obesity models, which may contribute distinct biological effects. A summary of some of the important secreted factors from BMAd and their mechanistic role in the BMAd‐interaction with diseased BM is shown in Table 1.

**TABLE 1:** Summary of mechanistic effects of secreted factors from BMAd niche.

BMAd secreted factor	Role	References
*Healthy BM*		
SCF	Supports stress and normal haematopoiesis	36, 37
Leptin	Increases proliferation of HSC and progenitors	39, 41
Adiponectin	Also supports HSC proliferation	42
Fatty acids	Support stress haematopoiesis	49
*Diseased BM*		
Fatty acids	AML:	
	‐Deliver energy substrate for tumour growth via blast‐expressed FABP4 transporter	66, 67, 68
	‐Engender chemotherapy‐resistant phenotype	78, 79
Leptin	ALL:	
	‐Supports ALL engraftment, may inhibit differentiation	64, 81
	MM:	
	‐Enhanced proliferation and niche homing, with reduced cell death	93
	‐Suppression of immune surveillance	96
	‐Promotes dexamethasone and bortezomib resistance	95
SDF1α	ALL:	
	‐Chemoattractant factor stimulating ALL migration to adipose tissue	87
	MM:	
	‐Increased invasiveness	108
Glutamine	ALL:	
	‐Resistance to asparaginase	89
Adiponectin	MM:	
	‐Induces MM‐cell apoptosis (negatively regulated in MM niche)	99, 100
Adipsin	MM:	
	‐Chemoresistance	101
Resistin	MM:	
	‐Chemoresistance	102
Exosomes	MM:	
	‐Chemoresistance	103

Abbreviations: FABP4: Fatty‐acid binding protein 4; HSC: Haematopoietic stem cell; SCF: Stem cell factor; SDF1α: Stromal cell‐derived factor 1α.

### AML

Mounting experimental evidence supports the premise that BMAd niches are key participants in AML leukaemogenesis and response to treatment (Figure 5A). A key mechanism is through metabolic reprogramming of AML cells facilitated by a dynamic and reciprocal interplay. BMAds generated from MSCs obtained from AML patient BM have been shown to promote survival and proliferation of primary AML through an intricate process that involves the release of free FA from BMAd, facilitated by AML‐induced deplidation of neighbouring BMAd and subsequent FA uptake into AML tumours to fuel tumour growth.^66^ FABP4 is the principal FA transporter/lipid chaperone implicated in this mechanism and increases are observed in AML cells upon exposure to BMAd stroma.^67^ A similar metabolic cross‐talk was also reported in in vitro models of human acute monocytic leukaemia cell lines using healthy human BMAd.^67^ Importantly, interfering with FABP4 or downstream metabolic consequences of FA translocation (e.g. inhibitors of FA oxidation) were shown to attenuate both AML survival^66^ and AML progression in vitro and in vivo.^68^ Aligned with those observations, strategies that reduce BMAd remodelling of larger marrow adipocytes into small marrow adipocytes; for example by interfering with BMAd remodelling leukaemia cell associated factor GDF‐15^69^ improves host survival in AML mouse models.^66^, ^70^ Thus, approaches that target the FA cross‐talk pathway at several entry points could present a novel approach to blocking pivotal functions of the BMAd niche in AML disease.

Whilst the BMAd morphology was not specifically assessed in these studies, the finding that small adipocyte abundance in the primary AML‐BM associates with a high risk of relapse and death in AML patients^71^ provides a clinical‐level significance to the notion that mechanisms that generate small marrow adipocytes, such as those arising from BMAd delipidation, have functional importance to AML survival. In a similar concept, a reduced adipocyte content in post‐therapy BM samples in AML patients correlates with extended remission duration,^72^ which is consistent with loss of a pro‐AML niche favouring better disease outcomes. Together these data point to the functional significance of BMAd depletion states in AML beyond simple histological observations.^73^

Apart from adipocyte delipidation‐driven alterations to the BMAd niche, there is also evidence that AML actively deregulates BMAd development through effects on the MSC stroma.^74^ AML‐MSC exhibit reduced intrinsic growth and colony‐forming unit potential and may also have a reduced capacity for adipogenic differentiation.^74^ Together with the finding that AML‐BM exhibits reduced MSC harvest efficiency,^75^ this points to cellular development of BMAds being disrupted in AML disease contributing to BMAd depletion states under these conditions.

Interventions that modulate BMAd tissue numbers have also undergone investigation with intriguing results. Boyd and colleagues identified that under conditions of human AML disease in vivo, normal myeloerythroid maturation becomes selectively disrupted due to the progressive depletion of the BMAd niche.^74^ Increases in BMAds, achieved by administration of a PPARγ agonist^76^ led to improved healthy haematopoiesis and concurrently reduced leukaemic colony‐forming cells and peripheral blood AML progression in mice.^74^ This proposes that pro‐adipogenesis therapy can simultaneously repress AML growth whilst maintaining healthy haematopoietic maturation. These apparently contradictory results with the pro‐AML inducing potential of BMAd described previously^66^ may reflect an important distinction between experimental strategies that target the global BMAd tissue versus a discrete BMAd‐associated molecule and highlight a need for better models to capture the full complexity of effects attributed to these niches.

Beyond BMAd support for the proliferation and survival of AML, the FA cross‐talk underlying this effect might also play a direct role in drug resistance.^77^ Support for this comes indirectly from studies showing that in a murine model of blast crisis chronic myeloid leukaemia, gonadal adipose tissue (GAT) serves as a distinct niche for a phenotypically distinct AML‐leukaemia stem cell (LSC) expressing lipid transporter CD36. CD36+ LSC shows a metabolic dependency for GAT‐associated FA release and their treatment‐resistant phenotype could be rescued by CD36 knockdown; demonstrating the specific role of FA pathways in AML drug resistance as previously suggested.^78^ Other adipocyte‐linked secretory factors regulating AML drug resistance include LEPR signalling, which reduces sensitivity to all‐trans retinoic acid and doxorubicin in Acute Promyelocytic Leukaemia cells by interfering with the signal transducer and activator of transcription‐3 (and mitogen‐activated protein kinase pathway).^79^

In summary, BMAd intersect with AML across multiple axes to enhance AML survival and proliferation and therapy resistance. Targeting the reciprocal relationship between BMAd and AML along with the FA interplay together with interventions that alter BMAd niche population numbers, may prove a valuable adjunct to anti‐AML therapy.

### ALL

Adipocytic tissues inclusive of BMAd are likewise implicated in the development and progression of ALL, although when compared to AML some principles are distinct (Figure 5B). In paediatric ALL, hypoadiponectinaemia, hyperleptinaemia and hyperresistinaemia occur at diagnosis and normalises in remission.^80^ The predicted consequence of this adipokine imbalance is loss of anti‐inflammatory (adiponectin) versus pro‐inflammatory (leptin and resistin) signals highlighting a possible role for AT inflammation in ALL pathogenesis. Functional support for this concept is best demonstrated for leptin, which is abundantly expressed in both human ALL and AML‐BM and in ectopic BM models biased towards BMAd predominance, where increases in leptin improve ALL homing and engraftment.^64^ Conversely, in mice, a reduction of leptin levels, both systemic and in the BM induced by repeated dietary fasting or a functional loss of LEPR signalling, was found to inhibit ALL development and trigger cell differentiation.^81^ Together, these data implicate leptin and LEPR pathway in ALL regulation and highlight how targeting leptin‐producing adipocytes and/or the leptin‐LEPR pathway (e.g. through medical mimics of the fasting response)^82^ may be beneficial in suppressing ALL growth).

At a cellular level, BMAd tissue niches have been shown to limit human B‐^83^ and T‐ALL^84^ repopulation in vivo, induce slow‐proliferation states and increase tumour dormancy in adipocyte‐rich tail‐BM models. Such outcomes associate with treatment insensitivity, particularly towards anti‐mitotic‐based chemotherapy.^84^ Concordant effects can be reproduced in vitro using a variety of adipocyte tissue niches strongly suggesting that ALL inhibitory outcomes are causally induced. These data support the concept that BMAd are a unique repressive niche for ALL permitting the establishment of dormancy, a feature intimately linked with treatment resistance. Notably, such results may also align with the inhibitory impact of fasting on ALL progression^81^ since fasting responses will be expected to increase BMAd numbers.^85^

Insights into the dormancy‐inducing properties of adipocytes have recently been elaborated by Heydt et al, who revealed adipocytes, both in vitro and in vivo, restrict protein synthesis in ALL cells.^83^ Protein translation elevation strategies using GCN2ib, targeting the major protein translation control hub EIF2α could partially rescue adipocyte‐induced translational repression in ALL cells, relieving ALL‐cell quiescence and reducing the treatment resistance conferred by adipocyte stroma. This finding opens up prospects for protein elevation strategies as a novel avenue for combating adipocyte‐driven niche resistance and counteracting a key ALL‐cell attribute linked with therapy failure.^86^

The multifarious routes by which adipocyte niches may confer treatment resistance present additional challenges. Studies that apply a combination of dietary‐induced obesity (adipocyte‐rich) models and in vitro co‐cultures of murine 3T3‐L1 adipocytes as well as adipocyte explants demonstrate the capacity of murine pre‐B ALL cells to migrate to subcutaneous and visceral (i.e. non‐BMAd) in vivo and in vitro,^87^, ^88^ an effect attributed to adipocyte secretion of the chemoattractant SDF1α^87^. Whilst obesity does not affect the time to development of ALL in vivo, it was associated with enhanced chemoprotective effects from vincristine.^88^ The adipocyte protective effect extended to dexamethasone, daunorubicin and nilotinib in vitro and was not dependant on cell to cell to contact but was linked with upregulation of anti‐apoptotic molecules Bcl‐2 and Pim‐2 in ALL cells and increased phosphorylation of Bad.^88^ Beyond chemoprotective effects, 3T3‐L1 adipocytes cause resistance to L‐asparaginase treatments via their capacity to secrete the metabolite glutamine, which is required for ALL‐cell survival and proliferation.^89^ Further follow‐up publications^90^, ^91^ focus on a single drug, daunorubicin, reporting that 3T3‐L1 adipocytes protect ALL cells from chemotherapy‐induced oxidative stress via soluble factors released upon tumour‐cell exposure. Critically, adipocytes also reduce available anthracycline levels through a process of drug sequestration and metabolic deactivation, which would predict lower active drug concentrations in the TME.^91^ Drug resistance conferred by adipocytes, therefore, encompasses multiple regulatory mechanisms. Of note, whilst adipocyte‐mediated FA cross‐talk has been shown to occur in ALL, its functional significance is less clear, particularly with respect to a clear impact on ALL‐cell proliferation.^83^, ^92^

### MM

As with AML and ALL, there is robust experimental data to demonstrate a bi‐directional interplay between MM cells and BMAds that supports MM proliferation and viability as well as generating a protective niche that allows evasion of anti‐tumour therapies (Figure 5C). An observation more specific to MM is that the BMAd niche plays a role in disrupting skeletal health, as well as the potential that BMAds reduce immune surveillance in MM by generating an immunosuppressive TME.

Adipokines in particular play a key role in the BMAd‐MM axis, supporting the survival and proliferation of MM cells. Seminal work here has shown that MM cells interact with BMAds to enhance their proliferation, reduce cell death and increase MM‐cell homing, driven at least in part by BMAd‐secreted leptin.^93^ Indeed, as with numerous other cancer types,^94^ leptin levels are higher in patients with MM compared to controls,^95^ supporting the concept of a leptin‐generated pro‐tumour environment. Leptin has also been proposed as an immune checkpoint in the MM niche due to its immunosuppressive effect on invariant Natural Killer T cells that favours MM growth.^96^ Targeting leptin, therefore, appears a rational approach to anti‐MM therapy and in the solid cancer arena this has garnered some interest, although a trial of metformin in breast cancer as a leptin‐reducing metabolic agent was unsuccessful.^97^ However, other approaches have been explored, and in the case of MM, LEPR antagonism has been proposed to restore immune surveillance in preclinical models.^96^

Contrasting the leptin effect, serum levels of adiponectin, an adipokine that is paradoxically downregulated in obesity, have been observed to be inversely proportional to MM risk,^98^ likely due to adiponectin's ability to activate MM apoptosis via activation of protein kinase A and AMP‐activated protein kinase pathway.^99^ Furthermore, MM cells are capable of downregulating BMAd adiponectin production directly via paracrine TNFα signalling,^100^ suggesting a mechanism by which MM reprograms BMAd niches to aid its survival. Adiponectin, therefore, has the potential to serve as a biomarker or therapeutic molecule in MM and a preclinical model in which acetyl‐coenzyme A carboxylase, an enzyme that maintains hypoadiponectinaemia, is inhibited has been described.^99^

BMAd‐linked secretory mechanisms also appear to play a significant role in MM chemoresistance. Again, leptin may have a key role here and it has been shown that exogenous as well as adipocyte‐derived leptin promotes resistance to dexamethasone and bortezomib in MM‐cell culture models.^95^ Concordant studies support the concept of BMAd‐driven chemoresistance to typical anti‐MM therapies that are affected by other adipokines including adipsin,^101^ resistin^102^ and BMAd‐derived exosomes.^103^ Identifying specific mechanisms of BMAd promoted drug resistance, therefore, offers the possibility of developing targeted therapies with the aim of deepening responses to conventional anti‐MM agents.

The remodelling impact of MM on the BMAd niche itself is complex and there is some variation in its characterisation between studies. MM may promote adipogenic differentiation and thereby increase TME BMAd content via secretion of DKK1 along with other cytokines.^104^, ^105^, ^106^ A predisposition towards adipogenesis exists in MGUS and smouldering myeloma (SMM) that can support disease progression through a coordinated provision of FA metabolic substrates.^107^ In keeping with this is the observation, the BM of MM patients contains increased numbers of preadipocytes and large mature adipocytes that functionally promote myeloma invasion through secreted factors including IL‐6, MCP‐1 and SDF‐1α.^108^ However, whilst not necessarily contradictory, other studies have observed that overall BMAT is reduced in MM BM samples^109^ and may contain fewer proliferative MSCs with the inhibited formation of small adipocytes.^110^ Indeed, MM cells have been shown to induce opposite effects on adipo‐lineage differentiation in a cell line‐specific manner.^111^ This range of data, with some inconsistencies between studies, provides a reflection on the complexity of assaying the heterogeneous BMME, suggesting that in future studies uniform criteria for enumerating and characterising BMAds will be essential.

Finally, BMAds appear to promote the skeletal pathology that is associated with MM. Liu and colleagues identified a MM‐driven, epigenetically mediated, reduction of BMAd PPARγ expression that drives osteoclastogenesis and inhibits osteoblastogenesis, ultimately resulting in bony disease.^112^ Importantly, disruption of the transcriptional repressor EZH2, led to increased PPARγ expression and reversed bone changes in mouse models.^112^ In parallel, MM cells were shown to directly induce a shift away from osteoblastogenesis by reprogramming BM‐MSCs,^113^ suggesting a cooperative process in which MM promotes adipo‐lineage MSC differentiation, which, in turn, promotes osteoclast‐induced bone damage.

## BMAds IN OTHER BM PATHOLOGY

Given the possible negative impact of adipocytes on haematopoiesis^32^ and the finding of BMAd accumulation in AA, alongside a reported association of BM‐MSC bias towards pro‐adipogenic outcomes in AA^114^ attempts to restore the unbalanced differentiation of BM‐MSC in AA have been investigated.^115^, ^116^ Arsenic trioxide, Wnt signal activation and microRNA‐204 interference are some of the strategies demonstrated experimentally to restore the unbalanced differentiation of BM‐MSC^117^ with improvements in haematopoietic function.^118^

Since BMAd accumulation is a defining feature of an ageing BMME, this has recently led to the exploration of its significance to clonal haematopoiesis. Recent insights using in vivo irradiation models to induce adipocytic hyperplasia demonstrate for the first time that the fatty BM provides a selective advantage to preleukaemic cells carrying DNMT3A‐R882H.^119^ IL‐6 was identified as one of the major players in the molecular mechanism that confers the fatty BM advantage specifically to R882H clones both in vitro and in vivo. Thus, BMAd may also be a sustaining factor in age‐associated premalignant states.

## INTERPLAY OF OBESITY, BMAd AND DISEASE

Epidemiological links between obesity, increased prevalence of haematological malignancy, haematological relapse and mortality lend support to the notion that BMAds, which are increased in number and volume in obesity, are active participants in haematological tumours.^120^, ^121^, ^122^, ^123^, ^124^, ^125^ Whilst retrospectively assessed correlative links between obesity and cancer outcomes should be interpreted with caution due to multiple potential confounding issues, there remain objective demonstrations to support a biological relationship between BMAds, obesity and adverse tumour outcomes. Thus, the striking clinical observations that obesity at diagnosis in children with ALL confers a worse event‐free survival rate and a higher likelihood for persistent minimal residual disease^126^, ^127^ require its biological and other underpinning drivers to be thoroughly explored. Key areas will be to address whether the causal link between obesity and worse disease‐specific outcomes may relate to whole‐body systemic factors or BMAd‐specific pathophysiology and to define to what extent does obesity‐associated BMAds create its own distinct biological context. Evidence for the latter comes from the observation that obesity‐associated adipocytes secrete greater levels of angiotensin‐II, which acts via receptors on MM cells to promote Acetyl‐CoA Synthetase 2 expression that, in turn, acts to enhance oncogenic activity of IRF2.^128^ Moreover, Bullwinkle and colleagues showed that abdominal fat‐derived adipocytes from obese patients generated a more tumorigenic microenvironment.^129^ Thus, whilst evidence is still sparse, there is the suggestion that obesity‐related BMAds may behave differently from non‐obese counterparts, and this may, therefore, represent an additional mode by which BMAd contribute to malignant outcomes.

## FUTURE PERSPECTIVES

The recognition that BMAd are a distinct entity with a range of metabolic, endocrine and secretory functions has gained wide‐scale acceptance. Whilst these key principles are well established, the full versatility of these tissues, their heterogenic nature and responses under different cancer contexts as well as their developmental origins are still not fully clarified. However, the demonstration that these tissues regulate multiple processes that impact haematopoietic health and disease underscores the crucial importance of resolving the complex and multifaceted nature of the BMAd niche in the future. Establishment of appropriate and more sophisticated model systems as well as innovations in spatio‐temporal‐mapping of the BMAd niche will be critical to resolving current controversies and for robustly determining mechanisms of BMAd involvement at both cellular and molecular levels. Such endeavours should now be considered a fertile field for future studies given prospects for a new cadre of niche‐based interventions.

## AUTHOR CONTRIBUTIONS

M.J.A., F.K., W.C. and B.P. wrote the manuscript. M.J.A., F.K. prepared figures and tables. B.P. conceived the study and supervised the work. All authors contributed to the article and approved the submitted final version.

## CONFLICT OF INTEREST STATEMENT

Not applicable.

## ETHICS APPROVAL STATEMENT

Not applicable.

## PATIENT CONSENT STATEMENT

Not applicable.

## PERMISSION TO REPRODUCE MATERIAL FROM OTHER SOURCES

Not applicable.

## CLINICAL TRIAL REGISTRATION

Not applicable.

## Data Availability

Not applicable.
